# Beneath the surface in autoimmune hemolytic anemia: pathogenetic networks, therapeutic advancements and open questions

**DOI:** 10.3389/fimmu.2025.1624667

**Published:** 2025-07-31

**Authors:** Alessandro Costa, Olga Mulas, Angela Maria Mereu, Mercede Schintu, Marianna Greco, Giovanni Caocci

**Affiliations:** ^1^ Department of Medical Sciences and Public Health, University of Cagliari, Cagliari, Italy; ^2^ Hematology Unit, Businco Hospital, ARNAS Brotzu, Cagliari, Italy

**Keywords:** autoimmune hemolytic anemias, cold agglutinin disease, warm autoimmune hemolytic anemia, pathogenesis, strategy, target therapy, complement system, immunotherapy

## Abstract

In recent years, the pathophysiologic framework of autoimmune hemolytic anemias (AIHAs) has evolved considerably, extending beyond the simplistic paradigm of antibody-mediated red blood cell (RBC) destruction, which is now recognized as a downstream consequence of a broader immune dysregulation. AIHA is fundamentally orchestrated by a complex interplay between innate and adaptive immune components, including autoreactive B and T lymphocytes, macrophages, and the reticuloendothelial system. Central to disease pathogenesis are two interrelated mechanisms: clonal B-cell expansion with autoantibody production and complement activation. These immunologic processes support the heterogeneity of AIHA, delineating distinct clinical entities such as warm AIHA, cold agglutinin disease/syndrome (CAD/CAS), and atypical variants, each characterized by specific therapeutic susceptibilities. Glucocorticoids remain the standard first-line therapy for warm AIHA; in contrast, CAD/CAS is increasingly managed with agents targeting B-cell function or complement activation, including rituximab and sutimlimab. However, therapeutic algorithms are rapidly shifting, particularly in the context of treatment-refractory disease. Emerging therapeutics targeting the classical complement pathway include novel anti-C1s monoclonal antibodies such as riliprubart, which exhibits an extended half-life due to enhanced affinity for the neonatal Fc receptor. Parallel strategies aim to disrupt B-cell receptor (BCR) signaling cascades, employing Bruton tyrosine kinase (BTK) inhibitors such as ibrutinib, spleen tyrosine kinase (SYK) inhibitors such as fostamatinib and sovleplenib, and phosphoinositide 3-kinase (PI3K) inhibitors such as parsaclisib. Collectively, these advances are reshaping the therapeutic landscape of AIHA toward a precision medicine model guided by mechanistic insights into disease biology. In this review, we delineate the evolving immunopathogenesis of AIHAs and examine emerging therapeutic strategies, integrating their underlying rationale, clinical data, and implications for future treatment paradigms.

## 1 Introduction

Autoimmune hemolytic anemias (AIHAs) are now recognized as complex immune-mediated disorders that extend beyond the traditional concept of isolated autoimmune erythrocyte destruction (1). The modern framework acknowledges this complexity, emphasizing the critical roles of both the innate and adaptive immune systems. Based on the isotype and thermal amplitude of the pathogenic autoantibody, these disorders are categorized into warm, cold, and mixed AIHA. However, the recognition of atypical forms, such as those with non-standard antibodies, negative direct antiglobulin test (DAT), or warm IgM involvement, has added complexity to diagnosis and classification (**Table 1**) (2). Thus, AIHAs figure as highly heterogeneous diseases, ranging from mild, stable cases to severe, relapsing, and potentially fatal ones (4). This heterogeneity is further illustrated by the variety of causes that can contribute to its onset. Genetic defects, alterations in immune response, neoplastic processes, infections, or drug exposure are commonly cited as pathogenic or predisposing factors (5).

**Table 1:** Clinical and laboratory characteristics of autoimmune hemolytic anemia subtypes (2, 3).

Characteristics	Warm AIHA (60-70%)	Cold AIHA		Mixed AIHA (5-10%)	Atypical AIHA (3-10%)
		CAD/CAS (20-25%)	PCH (1-5%)		
Antibody class	IgG	IgM	Donath-Landsteiner IgG	IgG and IgM	IgA, DAT-negative, warm IgM
Site of hemolysis	Extravascular (spleen)	Extravascular (mostly liver) and intravascular	Intravascular	Extra and intravascular	
Epidemiology	Prevalence of 17.01 per 100.000; median age at diagnosis of 58 years; M:F ratio 0.5-0.8	Incidence of 0.18-0.19 per 100.000 person-years; median age at diagnosis of 67 years; M:F ratio 0.7-0.8	Incidence of 0.04 per 100.000 person-years; predominantly pediatric onset (median age 5 years); M:F ratio 1.17	N/A	N/A
Underlying causes	LPDs, ADs (e.g. SLE); infections; drugs	In CAD there is evidence of low-grade B-cell LPD; in CAS, patients have associated conditions (e.g. infection, ADs; LPDs or other malignancies)	Infections (e.g. congenital or tertiary syphilis; measles; mumps); LPDs; ADs	LPDs, ADs (e.g. SLE); infections; drugs	IgA antibodies; RBCs-bound antibody below limit of detection; low-affinity AAb; warm-reacting IgM and monomeric IgM
Diagnosis	No cold associated symptoms with a DAT positive for IgG or C3d ± IgG when a clinically significant CA has been excluded	Monospecific DAT strongly positive for C3d (and negative or weakly positive with IgG) and a CA title ≥1:64 at 4°C	Hemolysis and a positive Donath-Landsteiner bithermic hemolytic test	DAT positive for C3d and IgG, a CA with title >1:64, thermal amplitude 4-30°C and evidence of a warm IgG antibody by IAT or IAT eluate	Extended DAT (“Super Coombs”) with specific anti-IgA or IgM antisera; LISS to identify low-affinity AAb; FCM to identify IgG bound to RBCs; polybrene to identify RBCs-bound antibody below limit of detection of standard DAT; column agglutination to identify low-affinity AAb

AAb, autoantibody; AIHA, autoimmune hemolytic anemia; ADs, autoimmune disorders; BM, bone marrow; CA, cold agglutinin; CAD, cold agglutinin disease; CAS, cold agglutinin syndrome; CS, cortisteroids; DAT, direct antiglobulin test; FCM, flow cytometry; IAT, indirect antiglobulin test; LISS, low-ionic-strength saline wash; LPD, lymphoproliferative disorder; PCH, paroxysmal cold hemoglobinuria; PB, peripheral blood; RBC, red blood cell; SLE, systemic lupus erythematosus.

Two interconnected immunopathogenic mechanisms are central to AIHA development. The first involves an autoreactive B-cell clone, sustained by a dysfunctional T-cell regulatory network, which produces autoantibodies that mediate primarily extravascular hemolysis via the reticuloendothelial system (2). The second crucial mechanism is complement activation, which exacerbates hemolysis, promotes cellular injury and sustains immune dysregulation. The resulting inflammation extends beyond red blood cell (RBC) destruction, affecting innate and adaptive immunity and increasing the risk of comorbidities such as vascular injury and thrombotic complications (3).

Emerging treatments targeting complement and B-cell clones are changing the AIHA treatment paradigm. As our understanding of AIHA deepens, it’s clear that a more comprehensive investigation of the connections between hemolysis, immune dysregulation, and inflammation is essential for improving patient outcomes and refining treatment strategies. This review examines the roles of innate and adaptive immunity and emerging therapies in AIHAs, aiming to enhance diagnosis, optimize treatment, and improve outcomes in these complex diseases.

## 2 Beyond the surface: divergent hemolytic pathways in warm and cold AIHA and the intricate cross-talk between complement and coagulation

### 2.1 Hemolytic mechanisms in warm AIHA

Warm AIHA, accounting for 60-70% of adult cases and approximately 50% of pediatric cases, is characterized by IgG autoantibodies with optimal binding at physiological temperature (∼37°C) (**Table 1**) (6). In the affected individuals, DAT is typically positive for IgG, and in most cases, the autoantibodies are polyclonal and directed against a range of erythrocyte antigens. The principal target is band 3 (SCL4A1), a major transmembrane anion exchanger. However, reactivity is frequently observed against other antigens, including Rh, Jk, Kell, Gerbich, Wright, glycophorin A and D, and Landsteiner-Wiener (7).

**Figure 1:**
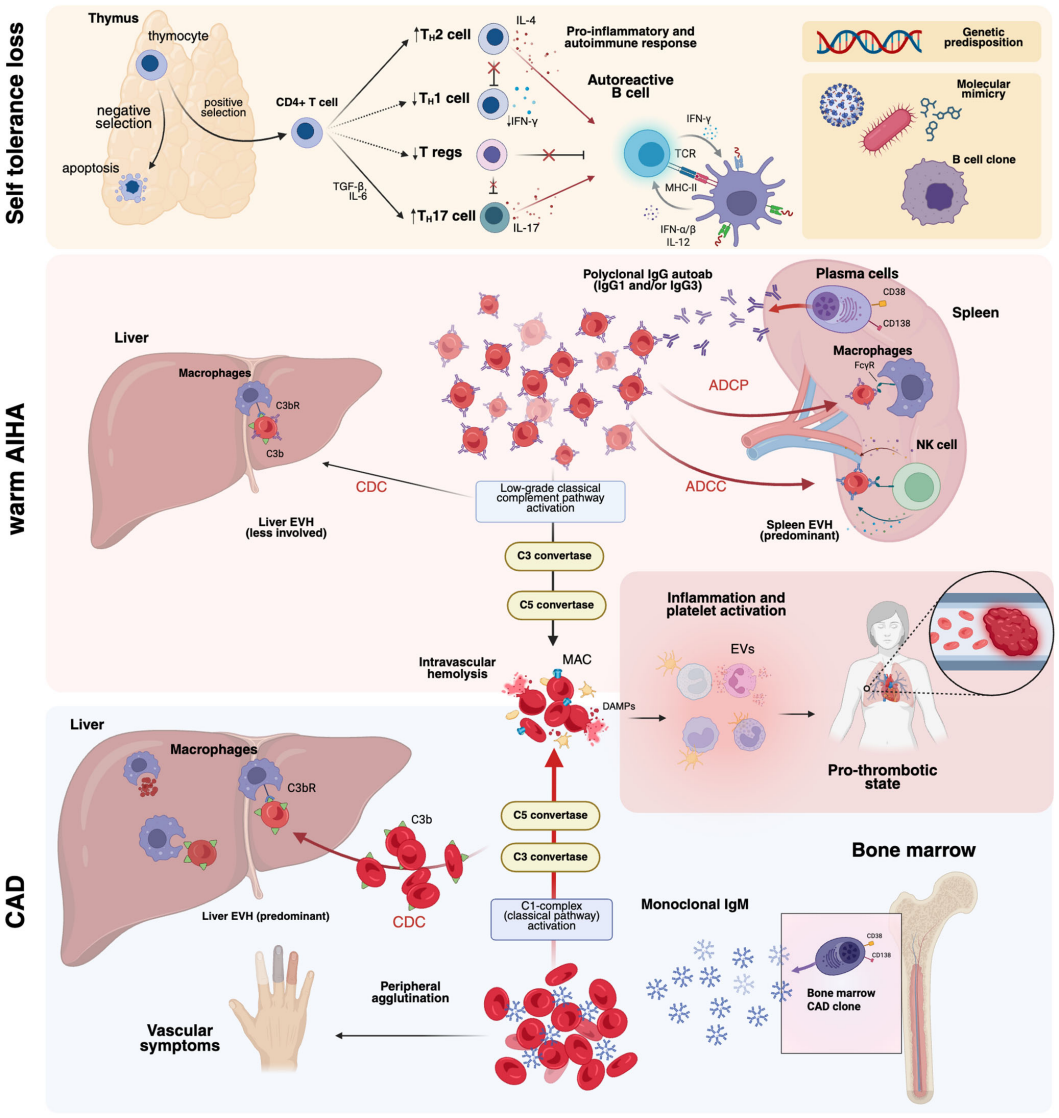
Overview of the pathogenesis of autoimmune hemolytic anemias (AIHAs). AIHAs result from a failure of immune tolerance, wherein defects in negative thymic selection permit the survival of autoreactive T lymphocytes. Dysregulation of T_H_1/T_H_2 homeostasis, coupled with genetic predisposition, contribute to aberrant B-cell activation. Molecular mimicry, often initiated by viral or bacterial infections, may further drive the clonal expansion of autoreactive B cells. In warm AIHA, autoantibodies primarily mediate EVH through splenic clearance via ADCP and ADCC, with a minor contribution from CDC in the liver. In contrast, in CAD, IgM autoantibodies bind erythrocytes at low temperatures, activating the classical complement pathway and leading to both IVH and EVH, primarily in the liver. ADCC, antibody-dependent cellular cytotoxicity; ADCP, antibody-dependent cellular phagocytosis; CAD, cold agglutinin disease; CDC, complement-dependent cytotoxicity. C3bR, C3b receptor; EVs, extracellular vesicles; EVH, extravascular hemolysis; IVH, intravascular hemolysis; MAC, membrane attack complex; TCR, T-cell receptor. Created in BioRender. Costa, A. (2025) https://BioRender.com/l08cmw5.

Hemolysis in warm AIHA predominantly occurs via extravascular mechanisms localized to the spleen and, to a lesser extent, the liver. Three principal immune effector pathways contribute to erythrocyte destruction (**Figure 1**): (1) antibody-dependent cellular phagocytosis (ADCP), (2) antibody-dependent cellular cytotoxicity (ADCC), and (3) complement-dependent cytotoxicity (CDC) (8). In the first two processes, the spleen serves as the primary site of extravascular hemolysis and the generation of autoreactive plasma cells, which sustain autoantibody production and contribute to hemolysis (9). Opsonized RBCs are recognized and phagocytized by red pulp macrophages via Fcγ receptors (FcγRs) IIa and IIIa (10). Among the IgG subclasses, FcγRs exhibit the highest affinity for IgG1 and IgG3 compared to IgG2 and IgG4 (11). Notably, splenic macrophages also express FcγRIIb, an inhibitory receptor whose activation may underlie the limited efficacy of intravenous immunoglobulin (IVIG) therapy in AIHA, as IVIG is thought to exert its effects, at least in part, through interactions with FcγRIIb (12). Natural killer (NK) cells and neutrophils, which express Fcγ receptors, can also mediate ADCC (13).

Although complement activation is generally less prominent in warm AIHA than in IgM-mediated cold AIHAs, IgG1 and IgG3 autoantibodies retain the capacity to activate the classical complement cascade through C1q binding. Following C1q engagement, conformational changes in the C1 complex (C1q, C1r, and C1s) initiate sequential proteolytic cleavage of C4 and C2 into C4a, C4b, C2a, and C2b, culminating in C3 convertase formation and downstream activation of the terminal complement cascade. This may result in variable degrees of intravascular hemolysis, although it typically remains a minor component compared to extravascular mechanism (14).

### 2.2 Hemolytic mechanisms in cold AIHA

Cold AIHA accounts for 20–25% of cases and is primarily driven by complement-mediated hemolysis. In these cases, DAT positivity is restricted to C3, and high-titer cold agglutinins exhibit optimal reactivity at low temperatures (typically 4–10°C) (**Table 1**) (15). A key distinction exists between cold agglutinin syndromes (CAS) associated with an underlying disorder and primary cold agglutinin disease (CAD), recently redefined as a clonal B-cell lymphoproliferative disorder in the 5th edition of the World Health Organization (WHO) classification (16). Specifically, CAD occurs in a clinically significant monoclonal gammopathy, where a clonal B-cell population produces auto-reactive IgM antibody. In contrast, in infection-associated CAS, IgM autoantibodies are usually polyclonal and less pathogenic than monoclonal ones (17).

In CAD/CAS, IgM autoantibodies primarily target the I antigen, which emerges on the erythrocyte membrane in adults following the enzymatic conversion of the fetal i antigen by β1,6-N-acetylglucosaminyltransferase (18). The pentameric nature of IgM promotes red cell agglutination, causing RBC aggregates that impair microcirculation in acral regions, where temperatures may fall within the thermal range permissive for agglutination even in the absence of environmental cold exposure (**Figure 1**) (19). Indeed, disease severity is primarily determined by the thermal amplitude of IgM rather than its serum titer, with a high thermal amplitude (>28°C) allowing for effective antibody binding even at normal body temperature (19). Notably, IgMκ binding to the erythrocyte surface in CAD induces agglutination and hemolysis by activating the classical complement pathway. Opsonization of RBCs by C3b promotes their extravascular clearance, primarily mediated by hepatic macrophages (20). However, terminal complement activation with membrane attack complex (MAC) C5b-C9 formation is generally absent during stable clinical phases and occurs predominantly during acute exacerbations or in severe disease (20, 21).

### 2.3 Hemolytic mechanisms in paroxysmal cold hemoglobinuria

Paroxysmal cold hemoglobinuria (PCH) is a rare and challenging entity within the spectrum of AIHA, accounting for less than 5% of cases and with an estimated annual incidence of 0.04 per 100.00 individuals (22). PCH is mediated by the Donath-Landsteiner antibody, a biphasic IgG that targets the RBC surface P antigen. Specifically, the Donath-Landsteiner IgG binds to its antigen at low temperatures and fixes early complement components C1, C4, and C2; upon warming to 37°C, the classical complement pathway is further activated, resulting in C3 cleavage and progression to terminal complement activation, which leads to acute intravascular hemolysis (22, 23). This pathogenic mechanism is unique among AIHA subtypes and contrasts with the primarily extravascular hemolysis observed in warm AIHA and CAD/CAS.

Historically, PCH was described as a chronic or recurrent complication of congenital or tertiary syphilis. The widespread use of penicillin led to a dramatic decline in syphilitic PCH, and the condition has since been reported predominantly in children following upper respiratory tract infections (24). However, recent epidemiological trends indicating a resurgence of syphilis underscore the need to reintegrate syphilis-associated PCH into the differential diagnosis of AIHA, including in adult patients (24).

### 2.4 Pro-thrombotic state in AIHA

Thrombosis is a major contributor to morbidity and mortality in AIHA, with both arterial and venous events occurring in 10–20% of cases (25–29). Retrospective data from 378 AIHA patients reported a 15% prevalence of venous thromboembolism (VTE), with warm AIHA and atypical variants carrying the highest risk (26). Notably, thrombotic rates appear consistent across different etiologies (30). Both a decade-long retrospective analysis and a Danish population-based study revealed a twofold increase in VTE risk in AIHA patients compared to non-AIHA controls (27, 28). Key risk factors include active hemolysis (hemoglobin ≤8 g/dL, elevated LDH), anemia severity, and prior splenectomy, emphasizing the role of hemolysis intensity in driving thrombosis (25, 29).

The classical coagulation model as a linear proteolytic cascade has evolved into a more integrated framework, incorporating the interplay between hemostasis, innate immunity, and the complement system (31). This revised paradigm is particularly relevant to AIHA, where intravascular hemolysis, complement activation, and inflammatory signaling converge to establish a prothrombotic state. While the mechanisms of hemolysis are well-characterized (32), emerging evidence provides novel insights. Mannes et al. (33) demonstrated that adenosine diphosphate (ADP), released upon MAC-dependent erythrocyte lysis, contributes to platelet activation and thrombotic propagation, in line with the pro-inflammatory role of damage-associated molecular patterns (DAMPs), such as heme (**Figure 1**) (31). Erythrocyte-derived extracellular vesicles (EVs), enriched in phosphatidylserine and tissue factor, have been implicated in AIHA-associated hypercoagulability. Barcellini et al. (34) found that EV levels were elevated in hemolytic patients, showing a correlation with the severity of anemia. Notably, patients with AIHA had much higher EV counts than healthy controls. Beyond hemolysis and erythrocyte-derived DAMPs, complement activation emerges as a key driver of the prothrombotic environment. A bidirectional interaction between complement and coagulation has been described (35), where complement activation enhances coagulation and vice versa through multiple mechanisms. C3 directly binds to fibrin, stabilizing clots and increasing resistance to fibrinolysis (36). Moreover, MAC activation on platelets at sublytic levels induces the externalization of procoagulant phospholipids, amplifying thrombin generation (37). Complement activation also drives the release of C3a and C5a, potent anaphylatoxins that recruit inflammatory cells and sustain thrombogenic inflammation (38). Conversely, thrombin cleaves C5 and C5b into non-canonical fragments that promote highly lytic MAC formation, linking coagulation and complement in a self-perpetuating cycle (35). These findings suggest that thrombotic risk in AIHA is not solely driven by hemolysis but reflects complex interactions between complement, platelets, and inflammation. This crosstalk underscores the rationale for complement-targeted therapies to mitigate thrombosis and improve clinical outcomes in AIHA.

## 3 Beneath the surface: self-tolerance breakdown and hidden drivers of hemolysis in AIHA

### 3.1 Mechanisms of self-tolerance loss

Historically, autoimmunity has been attributed to the interplay between genetic predisposition and environmental triggers, including infections, medications, or concomitant diseases, which together drive the emergence and clonal expansion of autoreactive lymphocytes (**Figure 1**) (39). In AIHAs, a known mechanism is molecular mimicry, where foreign antigens share structural or functional similarities with self-proteins, triggering cross-reactive immune responses. Notably, *Herpesviridae* (e.g., EBV, CMV, Varicella-Zoster virus) and *Poxviridae* exhibit a significantly higher degree of linear peptide homology with human proteins than other viral families, supporting their role in immune dysregulation (40). This concept gained attraction during the COVID-19 pandemic, as autoimmune cytopenias emerged in SARS-CoV-2-infected patients (41). Angileri et al. (42) identified an immunogenic epitope (LLLQY) shared between the RBC cytoskeletal protein ANK-1 and the SARS-CoV-2 spike glycoprotein, providing direct evidence of viral-induced AIHA (43). Beyond mimicry, viruses may further drive autoimmunity by exposing cryptic intracellular antigens upon cell lysis (44).

A similar mechanism may underlie drug-induced immune hemolytic anemia (DIIHA), where drug-dependent autoantibody formation occurs through immune-allergic hypersensitivity or non-immunologic protein adsorption (45). Among drugs commonly associated with AIHA, furosemide, antibiotics (e.g., amoxicillin, ceftriaxone, cefixime, cefpodoxime, ciprofloxacin, amphotericin B, sulfamethoxazole/trimethoprim, norfloxacin), azathioprine, NSAIDs (e.g., ibuprofen), and paracetamol have been implicated, with azathioprine carrying the highest associated risk (46).

While T-cell dysregulation has been a dominant focus, recent attention has shifted back to B-cell involvement, particularly the concept of a “forbidden clone”, an autoreactive B-cell population that escapes immune tolerance checkpoints, fueling persistent autoantibody production (47). Whether autoimmunity precedes or follows B-cell clonal expansion remains debated. Epidemiologic data show an increased risk of Non Hodgkin lymphomas (NHL) in patients with preexisting autoimmune diseases such as celiac disease and rheumatoid arthritis, supporting a two-hit model in which loss of immune tolerance precedes oncogenesis through chronic inflammation, persistent antigenic stimulation, and genetic susceptibility (48). Conversely, B-cell malignancies can drive autoimmunity via humoral and cellular dysregulation, as seen in hypogammaglobulinemia and altered regulatory T-cell subsets, underscoring the bidirectional link between lymphoproliferation and immune dysregulation (49). This is exemplified in AIHA associated with chronic lymphocytic leukemia (CLL), characterized by autoreactive polyclonal B cells alongside neoplastic monoclonal B cells (49).

The involvement of autoreactive lymphocyte clones is well established in warm AIHA and CAS; however, recent insights have refined the pathogenic understanding of CAD. Although overt lymphoid malignancy is typically absent, most CAD patients harbor a monoclonal IgMκ B-cell population, indicative of an underlying lymphoproliferative disorder (**Figure 1**). Randen et al. (50) delineated a clinically and immunophenotypically homogeneous entity in a cohort of 54 CAD patients. Bone marrow examinations revealed nodular infiltrates of mature B cells, lacking distinct morphological features, negative for the *MYD88* L265P mutation, and not fulfilling diagnostic criteria for lymphoplasmacytic lymphoma (50). This evolving paradigm redefines CAD as a distinct disorder, separate from other lymphoproliferative neoplasms, thereby sustaining its pathogenic framework and inclusion in the latest WHO classification.

### 3.2 Genetic background

Susceptibility to autoimmune diseases, including AIHA, is influenced by genetic variants that modulate immune responses. Given the critical role of the human leukocyte antigen (HLA) system, studies have focused on polymorphisms in specific HLA loci that alter the selection and activation of autoreactive T lymphocytes. Notably, HLA-B8 and BW6 alleles have been associated with an increased risk of AIHA, suggesting their involvement in the persistence of autoreactive lymphocyte clones (51). Additionally, specific immunoglobulin heavy-chain variable (IGHV) gene configurations appear to promote the selection of autoreactive B-cell clones (52, 53). For instance, IGHV4-34, IGHV4-31, IGHV3-23, and IGK3–20 gene rearrangements have been found in CAD patients (54–56). Moreover, genetic variants such as the G polymorphism in CTLA4 and the AG configuration in the lymphotoxin-α gene have been reported at higher frequencies in AIHA patients (57, 58).

In CAD, next-generation sequencing (NGS) has revealed gene mutations affecting B- and T-cell function. Notably, mutations in *KMT2D*, which encodes a histone methyltransferase involved in B-cell survival, differentiation, and homing, are present in 69% of cases. In contrast, mutations in *CARD11*, a key regulator of the NF-κB signaling pathway, are detected in 31% (59). Emerging candidate genes include *IGLL5*, whose precise role in B-cell development remains under investigation, and *CXCR4*, which plays a critical role in B-cell migration and trafficking (60). Additionally, decreased expression of *CR1*, a negative regulator of B-cell activation and differentiation, has been reported in CAD (56). The genetic contribution is even more pronounced in pediatric Evans syndrome (pES), a condition characterized by AIHA, immune thrombocytopenia, or autoimmune neutropenia (61). In a cohort of 80 pES patients, 40% were found to harbor mutations in immune-regulatory genes such as *TNFRSF6*, *CTLA4*, *STAT3*, *PIK3CD*, *CBL*, *ADAR1*, *LRBA*, *RAG1*, and *KRAS* (62).

Moreover, the role of polymorphisms in inflammatory cytokines has gained recent attention, further elucidating the complex genetic and immune dysregulation underlying AIHA. Specifically, Zaninoni et al. (63) identified single-nucleotide polymorphisms (SNPs) in genes encoding TNF-α, TGF-β1, IL-10, IL-6, and interferon γ (IFN-α), showing associations with disease severity and treatment response. In line with Pavkovic et al. (64), AIHA patients exhibited a lower frequency of the TNF-α -308 G/A polymorphism compared to controls, as well as a reduced genotypic frequency of TNF-α -308 G/A and TGF-β codon 25G/C. The genetic link with cytokines was further emphasized in pES, where PTPN2 haploinsufficiency has been identified, contributing to heightened sensitivity to cytokine signaling through the JAK/STAT pathway (65).

### 3.3 T cell dysregulation and polarization

Self-tolerance is a fundamental principle of immune homeostasis, essential for preventing aberrant autoreactive responses (**Figure 1**). In the thymus, central tolerance serves as the primary checkpoint for T lymphocyte selection and is coordinated by medullary thymic epithelial cells, cortical thymic epithelial cells, dendritic cells, and thymic B cells (66). These cells mediate antigen presentation and the clonal deletion of autoreactive T lymphocytes. Thymocytes expressing T cell receptors (TCRs) that fail to engage self-peptides presented via major histocompatibility complex (MHC) molecules undergo death by neglect due to insufficient survival signaling (67). Despite these mechanisms, up to 40% of autoreactive T cells and a comparable fraction of autoreactive B cells evade central tolerance (68, 69). Peripheral regulatory networks act as additional safeguards to limit their activation and expansion. These include T cell anergy, mediated by inhibitory receptors such as CTLA-4, clonal deletion via Fas-Fas ligand interactions, and suppression by CD4+/CD25+/Foxp3+ regulatory T cells (Tregs), which exert immunomodulatory effects through cytokines such as interleukin 10 (IL-10) and TGF-β (66, 70, 71).

T cells play a pivotal role in the pathogenesis of AIHA, contributing to the breakdown of immune tolerance and to the production of anti-erythrocyte autoantibodies. Unlike other self-reactive T cells, those targeting RBC antigens escape thymic negative selection. Instead, CD4+ recent thymic emigrants (RTEs) encounter RBCs in the periphery, where they modulate inhibitory receptor expression, particularly programmed death cell 1 (PD-1), and transcription factors associated with anergy, functional exhaustion, and regulatory differentiation (72). This dynamic suggests that tolerance to RBC autoantigens is primarily maintained through peripheral rather than central mechanisms.

In murine models, an imbalance between T helper 1 (T_H_1) and T helper 2 (T_H_2) subsets has been described, with a predominant T_H_1 response characterized by elevated IFN-γ production. Disease improvement in these models occurred in response to the T_H_2 cytokine IL-4 (73, 74). Conversely, human warm AIHA has traditionally been classified as a T_H_2-driven disorder, as indicated by elevated IL-4 levels and reduced IFN-γ secretion (75). Moreover, like other autoimmune diseases, AIHA seems to be marked by an imbalance between effector T cells and Tregs (76). The pathogenic role of T helper 17 (T_H_17) cells has been well documented in multiple inflammatory disorders (77), and an elevated presence of T_H_17 cells has been observed in AIHA patients, strongly correlating with disease activity (78). In murine models, dysfunction in CD4+/CD25+ Tregs impedes autoimmunity suppression, facilitating the production of erythrocyte autoantibodies (79). Recently, Ciudad et al. (80) observed a shift favoring T_H_17 polarization within T effector cells, associated with elevated serum IL-17 levels, aligning with findings by Xu et al. (78). Additionally, a reduction in circulating Tregs and decreased Foxp3 expression, a key transcription factor for Treg stability and suppressive function, were noted (80). Consistent with these observations, interference with RTE tolerance mechanisms using immune checkpoint inhibitors led to a concurrent decline in Foxp3+ Tregs and an increase in T_H_17 cells (81).

CD39 single-positive CD4+ T cells emerged as a predictive marker for AIHA development, suggesting their direct involvement in tolerance breakdown. CD39, an ectonucleotidase that hydrolyzes ATP and other extracellular nucleotides, modulates the immune microenvironment. Its dysregulation may contribute to the expansion of proinflammatory T cells, particularly T_H_17, while concurrently reducing regulatory populations, thereby promoting an autoimmune response against erythrocytes (81).

Increasing attention is given to the roles of follicular helper T cells (T_H_F) and follicular regulatory T cells (TFR) in autoimmune pathogenesis and erythrocyte hemolysis. T_H_F cells play a central role in complex regulatory events within the germinal center, including germinal center formation, B-cell support, isotype switching, high-affinity antibody production, and memory cell differentiation (82). In this setting, IL-6 and IL-21 contribute to T_H_F differentiation and function by upregulating the transcriptional repressor BCL6. In AIHA, a murine study reported increased levels of both T_H_F and TFR cells alongside elevated IL-21 and IL-6 levels. The pathogenic involvement of these subsets was further confirmed by the adoptive transfer of purified CD4+/CXCR5+/CD25− T_H_F cells from immunized mice, which induced autoantibody production in an AIHA murine model (83). Similarly, 24 patients with pES exhibited increased circulating T_H_F cells, heightened T-cell activation, and a reduction in naïve CD4+ T cells compared to healthy controls and patients with chronic immune thrombocytopenia (84). Post-activation exhaustion features were also noted, with upregulation of canonical checkpoint inhibitors, and a higher degree of β-chain oligoclonality in the TCR was observed in T_H_F cells compared to healthy controls.

### 3.4 Long-lived plasma cells and treatment failure

Following B-cell activation and differentiation within secondary lymphoid organs and inflamed tissues, a subset of plasma cells migrates to bone marrow niches, where they persist as long-lived plasma cells (LLPCs), comprising approximately 25% of the total bone marrow plasma cell pool (85). These CD38^+^/CD138^+^/CD20^-^ cells are responsible for sustained long-term antibody production, a function that can persist for decades (86). Their survival is regulated by stromal and immunoregulatory signals from dendritic cells, stromal cells, and regulatory T cells, which, through molecules such as CD80/86, CXCL12, and B-cell activating factor (BAFF), promote their persistence (86). At the molecular level, LLPCs overexpress anti-apoptotic genes (MCL1, BCL2, BCL-XL), making them highly resistant to immune-mediated depletion (87).

In warm AIHA, persistent autoantibody production is driven by autoreactive plasmablasts and plasma cells, with circulating plasmablasts indicating ongoing B-cell activation (9). Once settled in the spleen and bone marrow, LLPCs become refractory primarily to conventional therapies, including corticosteroids, rituximab, and other immunosuppressive agents targeting CD20-positive B cells. This limitation in therapeutic efficacy is particularly relevant in steroid-refractory or relapsing AIHAs, where ongoing autoantibody production by LLPCs sustains hemolysis despite the effective depletion of peripheral B cells (9). Moreover, it has been observed that B-cell depletion-induced alterations in the splenic microenvironment create a dependency of plasma cells on BAFF and CD4+ T cells. This suggests that the expression profile of LLPCs is influenced by signals derived from the splenic microenvironment (88).

## 4 Diagnostic approach and challenges in AIHA

### 4.1 Role and pitfalls of DAT

Precise classification of AIHA subtypes is essential to tailor treatment strategies and predict clinical outcomes. AIHA should be suspected in patients presenting with anemia and laboratory evidence of hemolysis, with the DAT serving as the primary diagnostic tool to confirm the immune-mediated nature of hemolysis (**Table 1**) (89, 90). The DAT detects immunoglobulin and/or complement bound to RBCs by using reagents that bind these components and induce visible agglutination *in vitro* (91).

The DAT-tube has traditionally been performed using polyspecific antiglobulin reagents, which yield semi-quantitative results without differentiating antibody isotypes or thermal amplitude. The composition of these reagents can vary significantly in the proportion of anti-IgG, anti-IgM, anti-IgA, and anti-complement components, often favoring anti-IgG (92). This approach can lead to false-negative results in cases involving IgA antibodies, low-affinity autoantibodies, or when the number of IgG molecules bound to RBCs falls below the assay’s detection threshold. The use of monospecific DAT enables accurate identification of specific autoantibody classes, thereby enhancing differential diagnosis and informing more precise therapeutic strategies (2, 15, 93) Additionally, employing low ionic strength solutions (LISS) or cold washes can overcome DAT negativity. Although less specific, more sensitive methods such as microcolumn and solid-phase antiglobulin tests allow for detection of low levels of IgG coating RBCs (93).

Among the most diagnostically challenging variants of AIHA, PCH warrants particular attention. The diagnostic process in PCH is often complex and far from straightforward (22). The DAT usually reveals isolated C3d positivity, which may also be observed in other complement-mediated forms of AIHA (22). Definitive diagnosis relies on the Donath-Landsteiner test, a technically demanding assay that is not routinely available outside of specialized laboratories (94). Therefore, clinicians should consider PCH in patients presenting with acute hemolysis and hemoglobinuria, especially when typical warm or cold autoantibody patterns are absent (22, 23).

### 4.2 Clinical assessment and diagnostic work-up

A detailed clinical history is essential to exclude alternative causes of hemolysis or confounding factors, such as recent transfusion reactions, drug-induced hemolysis, or glucose-6-phosphate dehydrogenase (G6PD) deficiency, which is more prevalent in specific populations (95). Evaluation should also include assessment for underlying disorders, particularly systemic autoimmune diseases (e.g., systemic lupus erythematosus) and lymphoproliferative malignancies like CLL or indolent NHL (90). Patient age can inform diagnostic considerations, with younger individuals more likely to present with underlying infections or autoimmune diseases, while older patients have an increased likelihood of harboring an active neoplastic disorder. Notably, infection screening is warranted when patients present with recent fever or respiratory symptoms; testing for EBV infection is particularly relevant in children and young adults (3). Signs such as weight loss, lymphadenopathy, hepatosplenomegaly, lymphocytosis, or cytopenias should prompt investigation for lymphoproliferative disorders.

Beyond standard hemolytic markers, extended immunohematologic and biochemical workup is essential to distinguish primary from secondary forms of AIHA (90, 96). Although this distinction is not immediately critical, since first-line treatment generally follows the same approach as for primary AIHA, it becomes clinically relevant when an underlying disorder requires targeted management (97). Autoantibody panels that include antinuclear antibodies (ANA), extractable nuclear antigens (ENA), anti-DNA, and antithyroid antibodies, should be considered in patients with suggestive clinical features such as arthralgias, serositis, or thyroid dysfunction. These tests may uncover an underlying connective tissue disease, guiding the use of immunosuppressants or targeted biologics (90). Moreover, in patients with a history of thrombotic events or recurrent abortion, antiphospholipid antibody testing is advisable. Viral serologies are also recommended (98). Serum protein electrophoresis, immunofixation, and quantitative immunoglobulin assays can help identify cases associated with plasma cell dyscrasias, such as Waldenström macroglobulinemia (99), or with primary immunodeficiencies (100). Bone marrow evaluation, including morphologic, histopathologic, immunophenotypic, and cytogenetic analysis, is essential for diagnosing AIHA associated with lymphoproliferative disorders, myelodysplastic syndromes, or marrow failure syndromes (90). This is particularly important at diagnosis in patients with CAD and in relapsed warm AIHA patients who are corticosteroid-refractory (90). Additionally, total-body computed tomography (CT) scanning is fundamental for staging and identifying lymphadenopathy or splenomegaly, which may indicate underlying lymphoid malignancies.

## 5 From biology to current and future therapeutic solutions in AIHA

Over the past decades, substantial advances in understanding AIHA pathophysiology have laid for a more tailored, subtype-specific therapeutic approach. However, despite these biological insights, current treatments still present important limitations. In warm AIHA, corticosteroids remain the mainstay of first-line treatment, with initial response rates of approximately 80% (97). Yet, sustained remission after tapering is achieved in only 30–40% of cases at one year (2, 4, 97). In CAD, corticosteroids are largely ineffective and should be avoided due to poor efficacy and frequent relapses after withdrawal. This reflects their inability to suppress autoantibody production in CAD. Likewise, splenectomy has no role in CAD due to hepatic rather than splenic clearance of erythrocytes (2, 4). Rituximab, targeting CD20+ B cells, has become the preferred second-line option in wAIHA and is increasingly used as a first-line treatment in CAD (97). Nonetheless, responses are often transient, and the efficacy of retreatment remains unclear. Other immunosuppressive agents, once used more broadly before the rituximab era, now play a limited role, often confined to later-line, steroid-sparing strategies, and lack strong prospective evidence (101, 102).

These limitations underscore a critical unmet need. The identification of B-cell dysregulation and complement activation as core pathogenic pathways has led to the development of novel targeted therapies. This progress reinforces the importance of precise disease classification, which now plays a central role in therapeutic planning and sequencing, marking a shift toward increasingly personalized and tailored management.

### 5.1 B cell-directed therapeutic strategies

**Figure 2:**
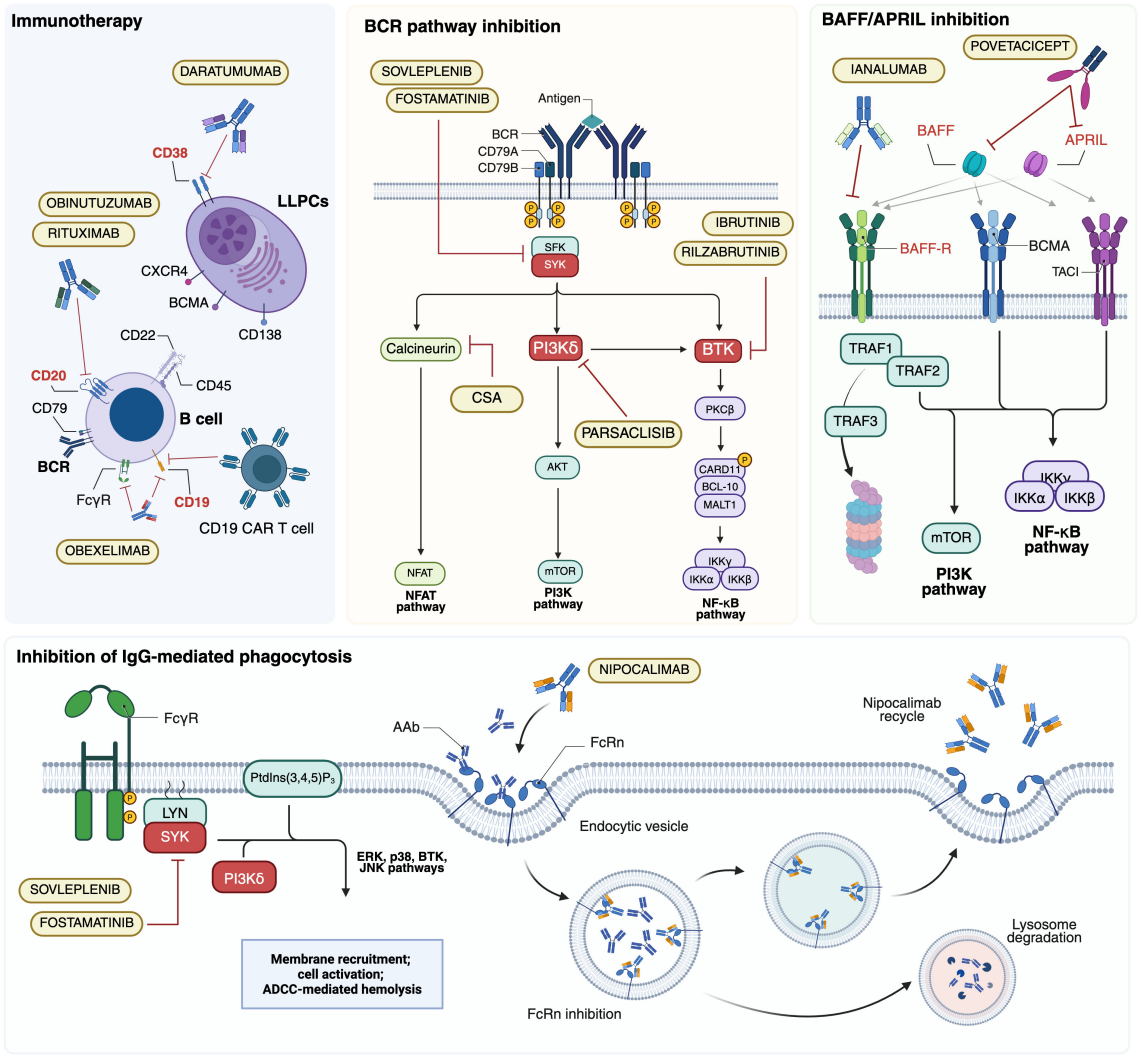
Major B-cell targeted therapy approaches investigated in autoimmune hemolytic anemias (AIHAs). AAb, autoantibody; ADCC, antibody-dependent cellular cytotoxicity; APRIL, a proliferation-inducing ligand; BAFF, B cell activating factor; BAFF-R, BAFF receptor; BCMA, B cell maturation antigen; BCR, B-cell receptor; BTK; Bruton tyrosine kinase; CAR T, chimeric antigen receptor T; CSA, ciclosporin A; FcγR; Fc γ receptor; FcRn, neonatal Fc receptor; LLPCs, long-lived plasma cells; PI3K, phosphoinositol 3-kinase; PtdIns(3,4,5)P3, phosphatidylinositol-3,4,5-trisphosphate; SYK, splenic tyrosine kinase; TACI, transmembrane activator and CAML Interactor. Created in BioRender. Costa, A. (2025) https://BioRender.com/kl39pwf.

Several therapeutic strategies that directly target B cells involved in autoantibody production (**Figure 2**) have been explored. These approaches aim to counteract relapse and treatment resistance, often driven by the persistence and escape mechanisms of autoreactive B cells at different stages of maturation. Among B-cell-directed therapies, targeting the B-cell receptor (BCR) signaling pathway is particularly relevant, given its central role in lymphoproliferative disorders and autoimmune diseases. The BCR regulates B-cell survival, proliferation, and activation through a complex intracellular signaling network involving Bruton tyrosine kinase (BTK), phosphoinositide 3-kinase (PI3K), and spleen tyrosine kinase (SYK) (103). The following sections explore these therapeutic strategies, evaluating their mechanisms of action, clinical efficacy, and limitations in the context of B cell-mediated autoimmunity.

#### 5.1.1 Anti-CD20 and anti-CD38 immunotherapies

Autoreactive CD20+ B lymphocytes play a central role in the pathogenesis of AIHAs, making them a crucial therapeutic target. Rituximab, a human IgG1κ MoAb originally developed for B-cell malignancies, was the first MoAb introduced in this setting (**Table 2**) (110). By binding to CD20 on the lymphocyte surface, rituximab induces apoptosis through ADCC and CDC mechanisms while modulating immune homeostasis by increasing Tregs and rebalancing the T_H_1/T_H_2 axis (104). In CAD, rituximab remains the preferred first-line therapy, inducing responses in approximately 50% of patients at one year, though durability remains a concern (111). Adding fludarabine enhances response rates but at the cost of increased toxicity, whereas bendamustine appears safer and potentially more effective, with response rates exceeding 70% (112, 113). In warm AIHA, rituximab is the preferred second-line treatment for steroid-refractory cases, achieving 70–80% response rates within 3–6 weeks (105, 114).

**Table 2:** Immunotherapeutic target and strategies in AIHA.

Target/drugs	Ref.	Study design	Disease, n. pts	Dosage	Efficacy	Safety
Anti-CD20 immunotherapy						
Rituximab	(104)	Phase 2, prospective multicentric study	CAD, n=27 pts	375 mg/m^2^ IV d1,8,15,22	14/27 pts improved anemia and hemolysis after 1 cycle; 13 pts were non-responder Median TTR: 1.5 mos (range 0.5–4 mos) Median DOR: 11 mos (range 2–42 mos)	No severe AEs reported
	(105, 106)	Phase 2, prospective multicentric study (NCT01345708)	CAD, n=14 pts; wAIHA, n=18 pts	100 mg fixed dose in d7,14,21,28	3-year OR: 100% in wAIHA vs 50% in CAD 3-y RFS: 76% in wAIHA vs 57% in CAD	No severe AEs reported
Obinutuzumab	(107)	Retrospective, single-center analysis	8 CLL pts; AIHA, n=4 pts	Over 6 cycles, at 100 mg d1, 900 mg d2, 1000 mg d8 and 1000 mg in subsequent cycles	CR: 100% TTR: 54.7 ds	Good tolerability; grade 3–4 neutropenia in one pt
Anti-CD38 immunotherapy						
Daratumumab	(108)	Retrospective observational, multinational analysis	19 pts; wAIHA, n=9 pts; cAIHA, n=7 pts (CAD, n=5 pts)	Variable, mostly 1080 mg SC x 4–8 wks or 16 mg/Kg IV x 4–8 wks (please, see Ref. 108 for specific dosages)	wAIHA: OR 50%; median Hb increase od 2.3 g/dL; DOR of 5.5 mos (range 2-12) cAIHA: OR 57%; median 3-mos Hb increase of 3.2 g/dL	Grade 2 infusion reaction in 2 pts with IV schedule.
Chimeric antigen receptor (CAR) T-cell therapy						
CD19 CAR-T (Inaticabtagene autoleucel)	(109)	Compassionate program and phase I clinical trial (NCT06231368)	Refractory AIHA pts failing ≥3 lines of treatment, n=8 pts	Single infusion of autologous CD19 CAR T-cells: 1×10^6^ cells/kg (compassionate use) or 0.5×10^6^ cells/kg (phase I, dose 1) after fludarabine (25 mg/m²/day, days -5 to -3) and cyclophosphamide (1 g/m², day -3)	CR achieved in 100% Median time to CR: 57 ds Median DFR: 6.3 mos	CRS reported in 5 pts ICANS reported in 1 pt

AE, adverse event; CAD, cold agglutinin disease; cAIHA, cold autoimmune hemolytic anemia; CLL, chronic lymphocytic leukemia; CR, complete response; CRS, cytokine release syndrome; ICANS, immune effector cell-associated neurotoxicity syndrome; IV, intravenous; DFR, drug-free remission; DOR, duration of response; OR, overall response; RFS, relapse-free survival; TTR, time to response; wAIHA, warm AIHA.

Recognizing the lower B-cell burden in autoimmune diseases compared to lymphoproliferative disorders, reduced-dose rituximab regimens have been explored. Studies investigating low-dose schedules (100 mg weekly for four weeks) reported response rates of 90%, with warm AIHA patients exhibiting superior responses and longer disease-free survival than cold forms (106, 115). Further insights come from a recent phase 2 pilot study assessing ultra-low rituximab doses (5 mg/m² every three weeks, 20 mg every four weeks, 50 mg every three months, and 100 mg every three months) in relation to CD20+ cell suppression (116). While nearly all patients achieved ≥95% CD20+ clearance after the first infusion, sustained depletion was not maintained. Instead, plasma rituximab levels above 0.4 μg/ml were required for complete peripheral CD20+ clearance.

Obinutuzumab, a second-generation anti-CD20 MoAb with enhanced direct cytotoxicity and ADCC has also been evaluated in AIHA, although data remains scarce. The most extensive analysis to date is a retrospective study of eight patients with CLL/SLL treated with obinutuzumab monotherapy for AIHA (n=4) or immune thrombocytopenia (ITP, n=4) (107). All AIHA patients achieved complete response, maintained at a median follow-up of 15 months. Ofatumumab, another anti-CD20 MoAb, has been used in isolated cases (117, 118). However, further studies are needed to establish the efficacy of anti-CD20 MoAbs beyond rituximab.

Considering the persistent autoantibody production by LLPCs, targeting CD38+ cells offers a novel therapeutic approach. Daratumumab, an IgGκ MoAb developed initially for multiple myeloma, was first reported by Scheutz et al. (108) in two cases of life-threatening warm AIHA post-stem cell transplantation, both successfully treated. At the same time, a third patient experienced a fatal relapse. Recent studies report a 50% response rate in warm AIHA with a median duration of 5.5 months, whereas in CAD, 57% of patients showed hemoglobin improvement, with clinical benefits such as reduced acrocyanosis (119). Additionally, due to CD38 expression on T lymphocytes, daratumumab may exert an immunomodulatory effect. A prospective analysis of two warm AIHA patients revealed a complete depletion of CD38+ T cells, impairing their activation and proliferation. Notably, in one patient, disease relapse coincided with the reappearance of CD38+ T cells, suggesting a link between therapeutic response and T cell repopulation.

Another anti-CD38 antibody of interest is isatuximab, which has a higher affinity for CD38 than daratumumab. A phase 1b/2 trial investigating its use in warm AIHA was initiated but was prematurely discontinued due to sponsor-driven strategic decisions (120).

Finally, obexelimab, a bifunctional, non-depleting humanized monoclonal antibody targeting CD19 and FcγRII, represents a promising therapeutic option for AIHA. Currently under investigation in a Phase 2 study (NCT05786573), its safety and efficacy are being evaluated in patients with warm AIHA. Interim results from six patients show a notable improvement in hemoglobin levels from baseline. Adverse events were reported in four patients, with three of 18 events considered drug-related. Further data are awaited to confirm these findings (121).

#### 5.1.2 BTK inhibitors

The BTK inhibitors have significantly improved treatment outcomes for patients with CLL/SLL and other related conditions (122). These agents are also being explored in autoimmune, allergic, and inflammatory disorders, including CAD. Notably, safety and efficacy data have emerged from a multinational retrospective analysis of 15 cold AIHA patients, 4 diagnosed with CAD and 11 with CAS, all receiving ibrutinib (123). Hemoglobin levels improved in all patients, with 12 achieving complete responses and one partial response, alongside transfusion independence and improved acrocyanosis. Adverse events were consistent with the known safety profile of ibrutinib. Rilzabrutinib, a reversible covalent BTK inhibitor, has been evaluated in a multicentric open-label phase 2b trial (**Table 3**). Preliminary results indicate a 64% response rate, with durable effects in 41% of patients (124). It is important to note that 86% of patients experienced adverse events, 18% of which were severe, with 45.5% deemed treatment-related. Further data is necessary to assess its safety and efficacy fully. Lastly, zanubrutinib, a next-generation, irreversible BTK inhibitor, is under investigation in a phase 2 trial (NCT05922839) for patients with warm AIHA and in a separate phase 2 trial involving CAD (NCT06067048).

**Table 3:** B cell receptor (BCR) pathway directed therapies in autoimmune hemolytic anemias (AIHAs).

Target/drugs	Ref.	Study design	Disease, n. pts	Dosage	Efficacy	Safety
BTK inhibitors						
Ibrutinib	(123)	Retrospective observational, multinational analysis	14 cAIHA pts; CAD, n=4 pts; CAS, n=11 pts	420 mg/die	12/13 achieved CR, with median Hb increase of 5.6 g/dL	Grade 1 AEs reported in 4 pts
Rilzabrutinib	(124)	Phase 2b, open-label multicenter trial (NCT05002777)	*Interim analysis*: 21 wAIHA pts and 1 aAIHA	400 mg BID x 24 wks	OR achieved in 14 pts (64%) including response† in 59% and CR in 14%	TRAEs reported in 10 pts (45.5%) No severe TRAEs
PI3Kδ inhibitors						
Parsaclisib	(125, 126)	Phase 2, open-label multicenter trial (NCT03538041)	25 pts; wAIHA, n=16; CAD, n=6; mixed AIHA, n=3 pts	1.0 mg QD (cohort 1), n=10 pts; 2.5 mg QD (cohort 2), n=15 pts; treatment continued for 12 wks	OR achieved in 16 pts (64%); CR‡ in 8 pts (32%) Higher responses in wAIHA vs other AIHA (75% vs 44.4%) Response rates ≥80% in pts who continued through the extension period, with no transfusion required Increased Hb level ≥2 g/dL from 16% in wk1 to 43.5% in wk12	TRAEs reported in 11 pts (44%) Severe TRAEs reported included diarrhea, CMV infection and psoriasis (each occurring in one pt)
SYK inhibitors						
Fostamatinib	(127, 128)	Phase 2, open-label multicenter trial (NCT02612558)	Primary or secondary wAIHA, n=26 pts	150 mg BID	Primary endpoint§ achieved in 11/24 pts (46%) by wk24; 25% achieved primary endpoint by wk6 15 pts (63%) improved Hb ≥1.5 g/dL from baseline	All-grade AEs reported in 100% of pts; grade 3 AEs reported in 46% of pts; Dose-reduction required in 3 pts (12%) for hepatic toxicity, neutropenia and diarrhea
		Phase 3 FORWARD trial (NCT03764618) Randomized 1:1 to FOS vs placebo	Primary or secondary wAIHA; FOS arm, n=45 pts; placebo arm, n=45 pts	100 mg BID, increased to 150 mg BID at wk4 if tolerated	Primary endpoint¶ achieved in 16 pts (35.6%) in FOS arm vs 12 (26.7%) in placebo arm (p=0.398) *Post-hoc* analysis reported higher responses in FOS arm vs placebo in USA, Canada, Australia and Western Europe (36% vs 10%, p=03) *Post hoc* reanalysis, excluding Hb values affected by salvage therapy, confirmed sustained response in 33.3% on FOS vs. 14% (6/43) on placebo (p = 0.0395).	Similar AEs incidence in FOS (93.3%) vs placebo (88.9%) arms. Severe AEs occurred in 33.3% of FOS arm vs 37.8% of placebo arm Five deaths (4.4% with FOS vs 6.7% with placebo) none treatment-related
Sovleplenib	(129)	Phase 2/3, randomized, multicenter trial (NCT05535933) Randomized 3:1 to sovlepenib vs placebo	Primary or secondary wAIHA; SOV arm, n=16 pts; placebo arm, n=5 pts	300 mg QD	Primary endpoint¶ achieved in 14 pts (67%), including 44% in SOV arm vs 0% in placebo arm by wk8. Sustained response by wk24 was 48% (10/21)	TRAEs reported in 14/21 pts (67%) One case of severe TRAEs (pulmonary embolism) The most frequent Grade-3 AE (≥2 pts) was anemia (19%), unrelated to treatment

AE, adverse event; APRIL, a proliferation-inducing ligand; BAFF, B cell activating factor; BTK, Bruton tyrosine kinase; CAD/CAS, cold agglutinin disease/syndrome; CMV, cytomegalovirus; CR, complete response; FOS, fostamatinib; OR overall response; PR, partial response; SOV, sovleplenib; TRAE, treatment-related adverse events; wAIHA, warm AIHA.

†Response was defined as increased Hb by ≥2 g/dL from baseline; CR was defined as Hb ≥11 g/dL in women and ≥12 g/dL in men with no evidence of hemolysis.

‡CR was defined as Hb value ≥12 g/dL not attributed to transfusion effect (i.e., no transfusions in the prior week); PR was defined as Hb value of 10–12 g/dL or ≥2 g/dL increase from baseline not attributed to transfusion effect.

§Primary endpoint was proportion of patients who achieved hemoglobin response by wk24; hb response was define as Hb level >10 g/dL and ≥2 g/dL higher than baseline, not attributable to blood transfusion or other medication.

¶Primary endpoint was the achievement of Hb ≥10 g/dL with ≥2 g/dL Hb increase during 24 wk-treatment period.

#### 5.1.3 PI3Kδ inhibitors

Another key target downstream of the BCR is PI3K, which plays an essential role in B-cell differentiation and survival (130). Parsaclisib, a selective and potent PI3Kδ inhibitor, has shown considerable efficacy in treating B-cell malignancies. Preclinical studies have demonstrated its ability to suppress B-cell proliferation, modulate regulatory T-cell homeostasis, inhibit CD8+ T-cell maturation, and influence immune compartment dynamics (125). In AIHA, preliminary findings from a phase 2 study of primary AIHA patients showed good tolerance and normalization of hemoglobin levels after 12 weeks of treatment with parsaclisib (126). Recent long-term data from 25 patients revealed early hemoglobin responses within the first week for half of the patients, with sustained improvements in the following weeks, leading to a 64% rate of partial or complete responses (131). Notably, higher responses were seen in patients with warm AIHA than those with CAD or mixed types. The drug was well tolerated, with 44% of patients experiencing treatment-related adverse events. A phase 3 randomized placebo-controlled trial is currently underway to evaluate its safety and efficacy in patients with primary warm AIHA (132).

#### 5.1.4 Inhibition of IgG-mediated phagocytosis

SYK is a key player in Fcγ-mediated phagocytosis of opsonized erythrocytes and B-cell activation/differentiation, making it an attractive therapeutic target. Its inhibition offers a twofold strategy: reducing autoantibody production and erythrocyte destruction. Fostamatinib, an oral SYK inhibitor, has received regulatory approval for ITP and has shown potential in warm AIHA. Based on preclinical efficacy in murine models (133), a Phase 2 study (NCT02612558) in warm AIHA reported a hemoglobin response in 46% of patients (**Table 3**) (127). The FORWARD trial further assessed fostamatinib in 90 patients with warm AIHA, randomized 1:1 to either placebo or fostamatinib (100 mg twice daily, escalating to 150 mg BID if tolerated) (128). Notably, two-thirds of patients had received at least one prior therapy, nearly all had been exposed to steroids, and half had been treated with rituximab. Although a greater proportion of fostamatinib-treated patients achieved hemoglobin improvement versus placebo, statistical significance was not reached. Regional differences were noted, with a more pronounced response in North American, Australian, and Western European patients, likely attributable to protocol deviations (including salvage steroid therapy during screening and two placebo-treated cases later determined unlikely to have warm AIHA) rather than true geographic differences (128). The drug was well tolerated, with diarrhea, hypertension, and fatigue as the most frequent adverse events.

A next-generation oral SYK inhibitor, sovleplenib, was evaluated in a Chinese Phase 2/3 study (NCT05535933) in 16 patients, with five receiving a placebo (129). By week 24, 67% of sovleplenib-treated patients achieved a hemoglobin response. Adverse events occurred in all patients, with 67% of them deemed drug-related. Further data are awaited to define its clinical role better.

FcRn inhibition, aimed at blocking IgG recycling and lowering circulating pathogenic antibodies, is also under investigation. Nipocalimab (M281), an anti-FcRn monoclonal antibody, has demonstrated promising preliminary results in a placebo-controlled Phase 1 trial on healthy volunteers (134). It is currently being evaluated in the Phase 2/3 ENERGY trial (NCT04119050), a randomized placebo-controlled study as an adjunct to standard therapy (135). Orilanolimab (SYNT001) and batoclimab (RVT-1401) are also under investigation for warm AIHA, though available data remain limited, necessitating further studies to assess their therapeutic potential (136).

#### 5.1.5 Inhibition of BAFF/APRIL signaling

BAFF (also known as BLyS, B lymphocyte stimulator) and APRIL (a proliferation-inducing ligand) are key TNF family members involved in B-cell survival, maturation, and activation (**Figure 2**). As previously highlighted, BAFF plays a role in B-cell repopulation following rituximab-induced depletion in warm AIHA, making it a target to prevent relapse via BAFF modulation (88).

Ianalumab (VAY736), an IgG1κ monoclonal antibody targeting BAFF-R, promotes B-cell depletion via ADCC and apoptosis. Preliminary data suggest that its depleting capacity may exceed that of anti-CD20 monoclonal antibodies. Based on these findings, the Phase 3 VAYHIA trial evaluates ianalumab in warm AIHA (137).

Povetacicept (ALPN-303) is a next-generation fusion protein designed to inhibit both BAFF and APRIL, acting through multiple receptors, including BAFF-R, TACI (transmembrane activator and calcium-modulating cyclophilin ligand interactor), and BCMA (B-cell maturation antigen). Preclinical models demonstrated that povetacicept reduces anti-RBC autoantibody production and antibody-secreting cells, depleting the spleen’s THF cells, B cells, and plasma cells. These effects were associated with increased hematocrit and a mild elevation in serum LDH (138). Further insights are expected from an ongoing Phase 1b open-label study in patients with autoimmune cytopenias, including warm AIHA and CAD (NCT05757570) (138).

#### 5.1.6 CD19 CAR T in AIHA

Chimeric antigen receptor (CAR) T cells have revolutionized lymphoproliferative malignancies management are now being explored in other hematologic and solid tumors. Emerging evidence also suggests efficacy in autoimmune diseases, including lupus, idiopathic inflammatory myopathies, and systemic sclerosis (139). Preliminary data from a median 6.8-month follow-up were recently reported in five patients treated with compassionate-use CD19 CAR-T therapy and three enrolled in a Phase 1 trial (NCT06231368) for refractory AIHA (109). Toxicity was manageable, with five patients experiencing grade 1 cytokine release syndrome (CRS) and one developing grade 1 immune effector cell-associated neurotoxicity syndrome (ICANS). Notably, all patients achieved complete responses.

### 5.2 Complement inhibition

**Figure 3:**
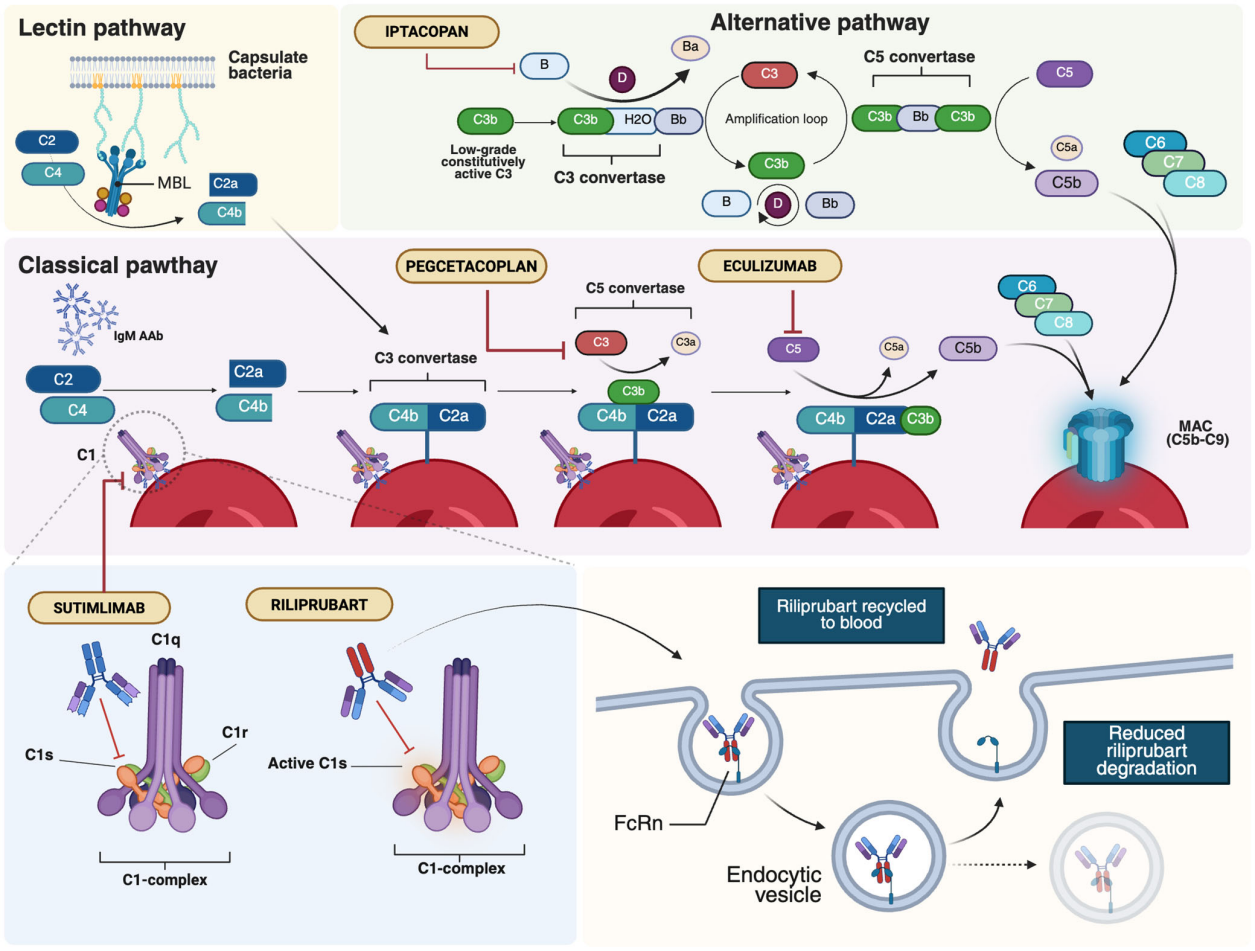
Major therapeutic targets within the complement system in AIHAs. AIHAs, autoimmune hemolytic anemias; AAb, autoantibodies; FcRn, neonatal Fc Receptor; MAC, membrane attack complex; MBL, mannan-binding lectin. Created in BioRender. Costa, A. (2025) https://BioRender.com/lj5h9fv.

Complement activation is a central driver of hemolysis in AIHA, particularly in CAD, where it mediates intravascular destruction and extravascular clearance of erythrocytes (2). Beyond hemolysis, excessive complement activation contributes to a procoagulant and inflammatory environment, exacerbating disease severity and increasing thrombotic risk. As shown in **Figure 3**, these pathogenic effects highlight the classical complement pathway as a critical therapeutic target, with emerging inhibitors showing promise in mitigating hemolysis and its systemic complications.

#### 5.2.1 C1 inhibitors

Given the central role of the classical complement pathway in CAD pathogenesis and the impact of C1 activation on extravascular hemolysis, selective inhibition of C1 has emerged as a promising strategy to modulate the disease while preserving other complement pathways.

Sutimlimab, a humanized IgG4 monoclonal antibody (MoAb) targeting C1s, is the first selective inhibitor of the classical complement pathway to receive approval for CAD (**Figure 3**) (140). By targeting an early step in the complement cascade, sutimlimab prevents C3b deposition on erythrocytes while sparing the alternative and lectin pathways. *In vitro* studies have demonstrated its ability to block C3 deposition on erythrocytes and inhibit the generation of anaphylatoxins, including C3a, C4a, and C5a, laying the foundation for clinical evaluation (20). In a phase 1b study with 64 healthy volunteers, sutimlimab infusions were well tolerated and showed a rapid concentration-effect relationship (141). The drug inhibited over 90% of classical complement activity within one hour of infusion, with doses ≥60 mg/kg every 14 days required to maintain stable inhibition. A phase 1b study in CAD patients revealed a significant increase in hemoglobin levels, although anemia recurred 3–4 weeks after discontinuation (**Table 4**) (141, 142). An extension of the follow-up to the study conducted by Jäger et al. (142) evaluated seven patients who had previously responded to the drug and were subsequently treated under a named patient protocol program. All patients exhibited a positive response to the treatment and maintained a transfusion-free status during the administration of sutimlimab (143). The safety and efficacy of sutimlimab were further assessed in the phase 2/3 CARDINAL trial, with the primary endpoint being the normalization of hemoglobin levels to ≥12 g/dL or an increase of ≥2 g/dL from baseline, without the need for RBC transfusions or the use of prohibited medications (144). A total of 24 CAD patients received sutimlimab (6.5 g for patients <75 kg, 7.5 g for those ≥75 kg) on days 0 and 7, followed by biweekly infusions for 26 weeks. Of these, 54% met the primary endpoint, and 83% achieved a stable increase of ≥1 g/dL in hemoglobin, with 71% not requiring transfusions between weeks 5 and 26. The most common adverse events included respiratory infections, diarrhea, and arthralgia, with no treatment discontinuations. No meningococcal events were reported. While reductions in D-dimer and thrombin-antithrombin complex levels were observed, conclusive evidence regarding the effect of complement inhibition on thrombotic prevention remains lacking. The two-year extension phase involving 22 patients from the part A study showed persistent improvements in hemolysis and anemia and positive effects on patient well-being (145, 146). Crucial findings also emerged from the phase 3 CADENZA trial, a randomized, placebo-controlled study in 42 CAD patients without a recent transfusion history, hemoglobin ≤10 g/dL, and at least one CAD-related symptom (147). Primary endpoints were the increase in hemoglobin level from baseline of ≥ 1.5 g/dL at treatment assessment timepoint (mean value from weeks 23, 25 and 26), absence of blood transfusions from week 5 to 26, avoidance of protocol-prohibited CAD medications from week 5 to week 26 (**Table 4**). Notably, 73% of the 22 patients receiving sutimlimab achieved the primary endpoint, compared to 10% of the 20 patients receiving placebo. Significant improvements in average hemoglobin levels and quality-of-life scores were observed in the sutimlimab cohort relative to the placebo group. As with the CARDINAL trial, the 1-year extension confirmed further improvements in hemoglobin levels from baseline (148). However, discontinuation of the drug was associated with a recurrence of hemolytic symptoms. A *post-hoc* analysis of combined phase 3 trial data stratified by baseline anemia severity included 24 patients from CARDINAL and 22 from the sutimlimab arm of CADENZA (153). Hemoglobin increases were proportional to baseline anemia severity, though differences between subgroups were not statistically significant. Real-world data remain sparse, and results from the multicenter, prospective CADENCE registry in CAD/CAS patients are eagerly awaited (154).

**Table 4:** Registered clinical trials exploring complement-inhibitions in AIHA.

Target/drugs	Ref.	Phase	Disease	Primary endpoint	n. pts	Efficacy	Safety
C1-inhibition							
Sutimlimab	141, 142	1b (NCT02502903)	CAD	Drug-related AE profile in healthy volunteers (phase 1a) and CAD pts (phase 1b)	10 pts	Median Hb increase was 1.6 g/dL by wk 1 and 3.9 g/dL by wk 6 Hb increased by ≥2 g/dL in 7/10 pts	No severe TRAEs
	143	NPP from phase 1b	CAD	Safety and efficacy of continuous long-term maintenance treatment	7 pts	SUT effect rapidly restored upon reinitiation after discontinuation Hb increased by initial median 7.7 g/dL to a median peak of 12.5 g/dL	62 AEs were reported, with 61/62 considered unrelated or unlikely related to sutimlimab 2 cases of BTH
	144-146	Phase 3 CARDINAL trial (NCT03347396)	Transfused pts with CAD	Part A – percentage of pts who avoided transfusion and achieving composite primary end point†	24 pts	13/24 pts (54%) reached the primary endpoint Hb increased by ≥1 g/dL in 7/24 pts Fatigue scores greatly improved	AEs reported in 22/24 pts (92%), 16 severe TRAEs in 7 pts 13 TRAEs in 9 pts No severe TRAEs No meningococcal infections reported
				Part B- long-term safety, tolerability and duration of response beyond 26 wks	22 pts	Sustained Part A response event at 2 years of follow-up	AEs reported in 22 pts; severe TRAEs reported in 2 pts (vitreous hemorrhage and viral infection) No meningococcal infections reported
	147, 148	Phase 3 CADENZA trial (NCT03347422) Randomized 1:1 to SUT vs placebo	Non-transfused CAD pts with Hb ≥10 g/dL	Part A – percentage of pts achieving composite primary endpoint ‡	SUT arm, n=22 pts vs placebo arm, n= 20 pts	Primary endpoint: 16/22 pts (72.7%) in SUT arm vs 3/20 pts (15%) in placebo arm Rapid increase in Hb level (within 3 wks) with 3-wks median increase of 2.7 g/dL from baseline	TRAEs in 8 pts (36%) in SUT arm vs 4 (20%) in placebo arm No meningococcal infections reported
				Part B – open-label extension for up to 1 y after the last pts completed Part A and a 9-wk washout period	39 pts	Effect of SUT were sustained for >79 wk on continued medication. Pts in the placebo arm switched to SUT after wk 26 (Part A) and rapidly achieved remission.	TRAEs were reported in 41% of pts. More frequently reported TRAEs were headache (10.3%) and cyanosis, fatigue, hypertension, injection site erythema, nausea and pyrexia (5.1% each) No meningococcal infections reported
Riliprubart	149	Phase 1b (NCT04269551)	CAD pts with Hb ≤11g/dL	Assess safety and tolerability of a single IV dose of riliprubart and dose-finding	12 pts; n=6 pts received 30 mg/Kg, and n=6 pts received 15 mg/Kg	Rapid improvement in Hb with mean Hb levels maintained at >11 g/dL from day 19 Hemolysis and anemia sustained improvement over 15 wks	No severe AEs More common AEs included: headache, acrocyanosis, rash, arthralgia and fatigue No meningococcal infections reported
C3-inhibition							
Pegcetacoplan	150	Phase 2 PLAUDIT trial (NCT03226678)	CAD and primary wAIHA	Assess safety of PEG	CAD cohort, n=13 pts; wAIHA cohort, n=11 pts	Median 48-wk Hb change from baseline was 2.4 g/dL for CAD pts and 1.7 g/dL for wAIHA	TRAEs reported in 9 pts (69.2%) in CAD pts and in 8 wAIHA pts (72.7%), more frequently injection site reactions No severe TRAEs No meningococcal infections reported
	151	Phase 3 CASCADE trial (NCT05096403) Randomized 2:1 to PEG vs placebo	CAD pts with Hb ≤9 g/dL	Hb level increase of ≥1.5 g/dL from baseline, maintained from wk 16-24 in absence of blood transfusion from wk 5-25	*Ongoing*		
C5-inhibition							
Eculizumab	152	Phase 2 trial (NCT01303952)	CAD	Difference in LDH level between the first and last day of treatment	13 pts	Median Hb increase from 9.35 g/dL to 10.15 g/dL; median LDH decrease from 572 U/L to 334 U/L 8 pts achieved transfusion independence	TRAEs reported in 4 pts No meningococcal infections reported

AE, adverse event; BTH, breakthrough hemolysis; CAD, cold agglutinin disease; IV, intravenous; PEG, pegcetacoplan; SUT, sutimlimab; TRAE, treatment-related adverse events; wAIHA, warm autoimmune hemolytic anemia.

†Composite primary endpoint in CARDINAL trial included: increase in hemoglobin level ≥2 g/dL from baseline or reaching a hemoglobin level ≥12 g/dL at the treatment assessment time point (mean of weeks 23, 25, 26); absence of transfusion from weeks 5 to 26; no use of protocol-prohibited medications.

‡ Composite primary endpoint in CADENZA trial included: increase in hemoglobin level from baseline of ≥1.5 g/dL at treatment assessment timepoint (mean value from weeks 23,25 and 26); absence of blood transfusions from week 5 to 26; avoidance of protocol-prohibited CAD medications from week 5 to week 26.

The second-generation C1 inhibitor riliprubart (SAR445088, BIVV020) is a humanized IgG4 MoAb that selectively inhibits the activated form of C1s, in contrast to sutimlimab, which targets both the active and inactive forms of C1s (**Figure 2**) (155, 156). Additionally, riliprubart is engineered with mutations that increase its binding affinity to the neonatal Fc receptor (FcRn), preventing lysosomal degradation and allowing for the recycling of the antibody within the system, thereby prolonging its half-life (157). A randomized, double-blind, placebo-controlled phase 1 trial was conducted to assess the safety, tolerability, and pharmacokinetics of riliprubart in 93 healthy subjects. The drug was administered either subcutaneously or intravenously in escalating doses, with both formulations demonstrating an acceptable safety profile. The subcutaneous formulation exhibited slow absorption and a prolonged half-life of 8 to 15 weeks (155). Based on these favorable pharmacokinetic properties, riliprubart was further evaluated in a phase 1b study (NCT04269551) involving 12 patients with CAD (**Table 4**) (149). Patients were administered a single intravenous dose of either 30 mg/kg or 15 mg/kg on day 1, followed by a 15-week monitoring period. The drug demonstrated a good safety profile, with no treatment-related adverse events leading to death or permanent study discontinuation. Furthermore, rapid improvements in hemoglobin levels and hemolysis biomarkers were observed in both dose groups within the first 30 days, with continued suppression of hemolysis throughout the 15-week study duration. An ongoing phase 1b extension study (NCT04802057) is evaluating the long-term safety and efficacy of riliprubart. Patients who completed the Phase 1a study and Part 1 of the LTS16637 study (receiving 600 mg subcutaneously every 4 weeks) transitioned to Part 2, where they received an intravenous regimen of 3.5 g every 12 weeks, with an additional loading dose on day 29. Preliminary data from four patients indicate sustained control of hemolysis and anemia, with hemoglobin and bilirubin levels maintained within normal ranges. The safety profile remains manageable, with no new or unexpected toxicities reported. Pharmacokinetic and pharmacodynamic analyses confirm predictable systemic drug exposure and sustained inhibition of the classical complement pathway, supporting the potential clinical applicability of the intravenous regimen (158).

Another investigated strategy involves administering a C1 inhibitor (C1-INH). Given its role as an endogenous complement regulator, C1-INH was evaluated in a phase 2 study (EudraCT2012-003710-13) in AIHA patients requiring transfusion support (159). Four intravenous doses were administered at 12-hour intervals. Hemoglobin levels temporarily increased post-transfusion but returned to baseline within 48 hours, suggesting the limited efficacy of this approach.

Finally, considering that over one-third of patients with warm AIHA exhibit complement activation, complement inhibitors may offer a promising therapeutic strategy, particularly in mixed forms with IgG and C3d positivity (2). In this regard, results are awaited from a phase 2 study (NCT04691570) evaluating subjects with warm AIHA showing evidence of complement activation or mixed forms, treated with ANX005, a recombinant anti-C1q IgG4 humanized MoAb.

#### 5.2.2 C3 inhibitors

Further downstream in the complement cascade, C3 inhibition has gained increasing interest due to its key role in CAD-related hemolysis. Pegcetacoplan, a polyethylene glycol-conjugated C3 inhibitor designed to prolong its half-life, exhibits high-affinity binding to both C3 and its activated fragment, C3b (**Figure 3**). The drug has already demonstrated robust efficacy and safety in paroxysmal nocturnal hemoglobinuria (PNH), securing Food and Drug Administration (FDA) and European Medicine Agency (EMA) approval (160). More recently, it has been evaluated in CAD in the phase 2 PLAUDIT trial (NCT03226678), which, unlike other studies, also included patients with relapsed, refractory, or treatment-intolerant warm AIHA (**Table 4**) (150). Overall, 13 CAD and 11 warm AIHA patients were enrolled and randomized to receive pegcetacoplan at either 270 mg/d (CAD, n=7; warm AIHA, n=5) or 360 mg/d (CAD, n=6; warm AIHA, n=6). In terms of efficacy, pegcetacoplan increased hemoglobin levels, with a median rise from baseline at 48 weeks of 2.4 g/dL in CAD and 1.7 g/dL in warm AIHA. In the warm AIHA cohort, hemoglobin levels increased until week 8, then plateaued. The drug was well tolerated, with diarrhea, headache, hypertension, nausea, and vitamin B12 deficiency being the most frequently reported adverse events. Injection-site reactions occurred in 46.2% of patients, and seven developed infections. One AIHA-related death was reported, attributed not to the drug but to the patient’s complex clinical management due to other complications. Although ten patients experienced at least one serious adverse event, none were considered treatment-related. The phase 3 CASCADE trial, a randomized, placebo-controlled study (NCT05096403), is ongoing, with eagerly awaited results expected to further define this agent’s efficacy and safety profile (151).

#### 5.2.3 Other complement target and inhibition strategies

Another explored inhibitor is eculizumab, an anti-C5 MoAb known for revolutionizing the therapeutic landscape of PNH, another form of complement-mediated, DAT-negative hemolytic anemia (**Figure 3**) (161). Building on promising off-label reports from individual case studies (162–164), the phase 2 DECADE trial, conducted in 13 CAD patients, demonstrated a reduction in transfusion requirements, although the median hemoglobin increase remained modest, likely due to persistent C3b-mediated opsonization and ongoing extravascular hemolysis (152). Eculizumab has also shown clinical efficacy in a pediatric case of PCH in a 4-year-old child who was refractory to corticosteroids but experienced marked clinical improvement following a single dose of the anti-C5 antibody (23).

More recently, factor B inhibition has garnered interest, mirroring therapeutic advancements in PNH. Iptacopan, an oral selective factor B inhibitor, has shown efficacy in PNH and other complement-mediated disorders (**Figure 3**) (165). The drug is currently under evaluation in CAD (NCT05086744), aiming for a hemoglobin increase of at least 1.5 g/dL from baseline. An interim analysis of 10 enrolled CAD patients reported that the primary endpoint was met in 50% of cases, with a mean hemoglobin increase of 1.8 g/dL and positive trends in other biomarkers and FACIT-fatigue scores (166). The drug was well tolerated, with no reported adverse events deemed treatment-related.

## 6 Conclusion and open questions

The landscape of AIHA has evolved, revealing a complex interplay between innate and adaptive immune mechanisms. While recent advances have led to more targeted therapeutic strategies, uncertainty persists, particularly in patients with refractory or relapsing disease. Moving forward, key challenges include refining the management of refractory cases, identifying reliable biomarkers to guide treatment selection, and addressing the underlying mechanisms of therapeutic resistance and autoantibody persistence. A deeper understanding of these factors is crucial, not only to enhance treatment effectiveness but also to prevent chronicity. The future of AIHA lies in integrating novel therapeutic approaches with personalized care to address ongoing clinical challenges and improve patient outcomes.
